# Helium spin-echo as a surface-sensitive probe of vibrational energy dissipation

**DOI:** 10.1039/d5fd00162e

**Published:** 2026-06-08

**Authors:** Anton Tamtögl, John Ellis, William Allison, Agata Sabik, Grażyna Antczak, A. P. Jardine

**Affiliations:** a Institute of Experimental Physics, Graz University of Technology Graz Austria tamtoegl@tugraz.at; b Cavendish Laboratory J. J. Thompson Avenue Cambridge CB3 0HE UK; c Institute of Experimental Physics, University of Wrocław Wrocław Poland; d National Synchrotron Radiation Centre SOLARIS, Jagiellonian University Krakow Poland

## Abstract

Understanding vibrational lifetimes at surfaces is central to advancing our knowledge of thermal transport, energy dissipation, and nanoscale friction. While phonon lifetimes in the bulk are routinely accessed *via* inelastic neutron scattering or optical phonon modes *via* high-resolution Raman spectroscopy, the direct measurement of lifetimes for low-energy surface acoustic phonons, particularly at finite wavevector, remains a major experimental challenge. This is due to the extremely narrow linewidths involved, corresponding to picosecond lifetimes and requiring µeV energy resolution. Here, we demonstrate how helium spin-echo (HeSE) spectroscopy overcomes this limitation, enabling direct access to the intrinsic linewidths and lifetimes of surface vibrational modes. For Ag(001), we map the full dispersion of the Rayleigh wave. Temperature-dependent measurements at finite wavevector reveal its weak anharmonicity and allow extraction of its intrinsic linewidth from the associated broadening. This corresponds to a phonon lifetime of ≈29 ps at 0 K and a propagation length of ≈44 nm, indicating coherence over tens of nanometres despite electron–phonon and defect-induced scattering. In a complementary application, we explore the vibrational dynamics of organic adsorbates, using cobalt phthalocyanine (CoPc) on Ag(001) as a model system. Low-frequency frustrated translational modes of the adsorbed molecules illustrate HeSE’s capability to probe vibrational damping in complex adsorbate–surface systems. The observed linewidths reflect enhanced dissipation arising from intermolecular interactions and coupling to the substrate. These findings establish HeSE as a sensitive probe of vibrational energy dissipation at hybrid organic–metal interfaces. Taken together, these capabilities open new avenues for the quantitative study of phonon lifetimes and linewidths in complex and emergent material systems, including 2D heterostructures and unconventional superconductors, where vibrational dynamics and their coupling to other degrees of freedom play a decisive role.

## Abstract 2

Helium spin-echo spectroscopy can directly quantify linewidths and lifetimes of surface vibrational modes, from Rayleigh waves on Ag(001) to vibrations of organic molecules, giving direct access to energy dissipation and coherence at interfaces.

## Graphical abstract

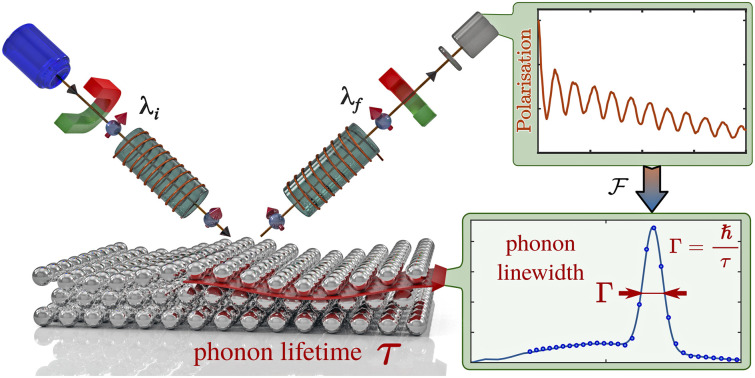

## 1 Introduction

Energy dissipation at surfaces governs key processes including chemical reactions, materials growth, and a broad range of physicochemical transformations central to surface and interface science.^1–5^ Despite its fundamental importance, dissipation remains poorly understood at low energies and on the atomic scale,^2,6–8^ where the coupling between adsorbates, substrates, and their elementary excitations gives rise to complex relaxation pathways. An understanding of phonons, the quantised modes of lattice vibrations, is therefore essential not only for elucidating energy dissipation at surfaces but also for describing the broader landscape of physicochemical processes they govern. Phonons underpin heat transport, phononic engineering, and nanoscale acoustics,^9–14^ and they mediate the dynamics of other quasiparticles including electrons, magnons, plasmons, and excitons. Their coupling to these excitations shapes transport properties, topological surface states,^15,16^ electrical conductivity,^17^ superconductivity,^18–22^ magnon–phonon interactions,^23^ plasmon–phonon hybridisation,^24,25^ and exciton dynamics.^26^ A fundamental descriptor of these interactions is the phonon linewidth, which reflects the finite lifetime of each vibrational mode and thus encodes the intrinsic scattering processes that characterise a given material surface.

In an ideal harmonic crystal with no scattering, phonon lifetimes would be infinite. However, in real materials, anharmonicity, electronic coupling, impurities, and interactions with other quasiparticles give rise to a measurable energy broadening. For low-energy acoustic modes, these linewidths are typically extremely small, corresponding to picosecond lifetimes, and demand sub-meV, often µeV-level, energy resolution for direct experimental access.^10^ Measuring such narrow linewidths at surfaces is even more challenging: surface modes generally occur at lower energies than their bulk counterparts and, crucially, require genuinely surface-sensitive probes.

Hence, while bulk phonon lifetimes are routinely accessible *via* high-resolution inelastic neutron scattering, which has been instrumental in identifying anharmonicity, mode softening, and lifetime variations near structural or superconducting phase transitions,^10,27,28^ experimental information of surface phonon linewidths remains scarce. Classical helium atom scattering (HAS) can resolve low-energy surface modes and has provided measurements of temperature-induced anharmonic shifts for selected systems,^29,30^ but its energy resolution is insufficient to determine intrinsic linewidths for long-wavelength acoustic modes. Consequently, surface phonon studies have largely focused on higher-energy excitations at the Brillouin-zone boundary,^31^ or, for optical modes, on Raman-accessible frequency ranges.^32–36^ Overcoming these limitations, in order to directly measure acoustic phonons close to the Brillouin-zone centre, is essential for establishing a broader picture of phonon-mediated behaviour,^37–40^ and for providing a quantitative link to the microscopic scattering mechanisms that govern both energy dissipation and the wider set of physicochemical processes at surfaces.^41^

Helium spin-echo (HeSE) spectroscopy resolves this limitation: by measuring the intermediate scattering function in the time domain, HeSE achieves energy resolutions down to tens of µeV, enabling direct access to intrinsic linewidths of surface phonons even in the low-energy, long-wavelength regime.^42^ Its use of neutral He atoms with thermal energies of ∼10 meV provides a non-destructive, chemically inert probe ideally suited for sensitive surfaces.^30,43^ Because He atoms interact primarily with the surface charge density corrugation,^44^ inelastic scattering directly reflects the phonon-induced modulation of the charge density, offering insight into electron–phonon (e–ph) coupling at surfaces.^44–46^ While earlier work has demonstrated that HeSE is capable of resolving phonon linewidths on metal surfaces,^14,47,48^ we introduce the first systematic methodology that enables both the determination of anharmonicity and the extraction of intrinsic lifetimes for low-energy surface phonons. In a complementary direction, vibrational dynamics at organic–metal interfaces are of increasing interest. As a model system, cobalt phthalocyanine (CoPc, C_32_H_16_CoN_8_) offers a prototypical organic adsorbate relevant to organic electronics and sensing applications,^49–52^ and even proposals for molecular quantum information technologies.^53,54^ Access to the linewidths and lifetimes of low-frequency frustrated modes in such adsorbates provides a new route to understanding vibrational damping, molecule–substrate coupling, and the microscopic mechanisms governing energy dissipation in hybrid organic–metal systems.

In summary, in this work we demonstrate the capability of HeSE to address these outstanding challenges: we provide temperature-dependent measurements of the Rayleigh wave (RW) on Ag(001), quantify its anharmonicity and intrinsic linewidth, determine its intrinsic lifetime and propagation length, and extend the method to probe molecular vibrational dynamics in CoPc/Ag(001). These results open new avenues for exploring phonon lifetimes across emerging material platforms, including 2D heterostructures, correlated materials, and systems exhibiting unconventional electronic phases. Finally, an improved understanding of vibrational lifetimes at surfaces is also of growing relevance for fields such as ultrafast energy dissipation, models of electron–lattice relaxation,^7^ and nanoscale energy accommodation, where quantitative knowledge of linewidths is essential.^55,56^ New experimental approaches^57^ and, in particular, theoretical approaches, including fully quantum mechanical scattering theories,^58,59^ have further highlighted the need for reliable experimental linewidths, particularly in systems where surface defects^48^ or heterogeneous interfaces play a significant role. The same applies to 2D materials such as graphene, in which both bulk and surface phonons influence e–ph coupling and transport properties.^60,61^

## 2 Experimental methods

The measurements were performed on the Cambridge helium-3 spin-echo (HeSE) spectrometer, which employs a nearly monochromatic ^3^He beam in a fixed 44.4° source-target-detector geometry. Detailed descriptions of the instrument can be found in ref. 62–64. The Ag(001) single crystal substrate was mounted on a cartridge allowing for radiative heating and cryogenic cooling down to 135 K. The temperature was monitored using a chromel–alumel thermocouple. Standard sputter–anneal cycles (Ar^+^, 0.8 keV, 10 µA; annealing to 800 K) were employed to prepare a clean and well-ordered surface, as confirmed by helium diffraction and reflectivity measurements.

**Fig. 1:**
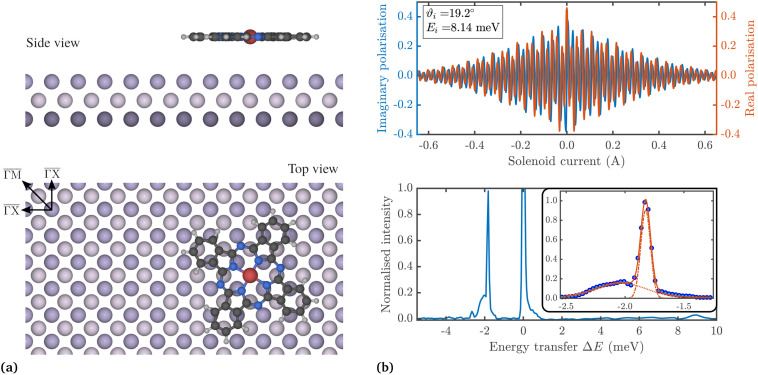
(a) Illustration of the surface structure with CoPc adsorbed on Ag(001). Schematic of the Ag(001) surface with a CoPc molecule (C_32_H_16_CoN_8_) adsorbed on the right-hand side. (b) ISF measurement of pristine Ag(001) and corresponding energy spectrum with RW and longitudinal resonance (LR) modes fitted. The upper panel shows an ISF for a typical example measurement of Ag(001) at 135 K, while in the lower panel, the data has been converted to the energy transfer scale. The inset shows the fitting of the Rayleigh (RW) mode and the longitudinal resonance (LR) with two Gaussians.

For measurements of adsorbate diffusion and vibrations, CoPc molecules were deposited from a home-built Knudsen cell containing crystalline powder (Sigma-Aldrich), held at 620 K, while the substrate was maintained at 350 K. CoPc adsorption proceeds *via* the formation of well-ordered islands on Ag(001), beginning near 0.8 monolayer (ML) coverage,^65,66^ with the molecules arranged in a (5 × 5) superstructure.^67^ Below this threshold, mobile CoPc molecules coexist in a two-dimensional gas phase.^65^ CoPc adsorption occurs in a planar adsorption geometry on Ag(001), as shown in Fig. 1(a).^67^ The evolution of surface coverage was monitored through the attenuation of the specular helium beam during deposition.^68^ Here, the HeSE technique has traditionally been mostly employed to investigate fast surface dynamics of atoms and molecules in thermal equilibrium,^69–73^ but it also enables the direct measurements of adsorbate vibrational modes, as demonstrated in the present study.

HeSE provides access to the intermediate scattering function (ISF) as a function of spin-echo time *t*_SE_ by controlling the nuclear spin precession of the ^3^He beam *via* magnetic fields generated by solenoid currents before and after surface scattering (see also SI).^63,69,71,74^ Vibrational information is extracted from spin-echo measurements across a wide range of *τ*_SE_, capturing both the real and imaginary components of the beam polarisation. Oscillatory features in the polarisation indicate periodic motion of surface atoms or adsorbed species, and correspond to distinct vibrational energies.

The measured polarisation as a function of solenoid current, *P*(*I*), reflects contributions from both inelastic vibrational excitations and quasi-elastic scattering due to adsorbate diffusion. While in practice multiple vibrational modes usually contribute, the essential behaviour can be illustrated using a simplified model with a single vibrational frequency:1
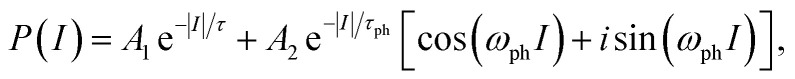
where *τ* describes the decay due to diffusive motion, *ω*_ph_ is the phonon frequency, and *τ*_ph_ denotes the phonon lifetime. In the present context, we are not concerned with the quasi-elastic (diffusive) contribution, which gives rise to a monotonic decay; our focus is on the inelastic component, manifested as damped oscillations in the polarisation arising from phonon excitations. Fig. 1(b) shows a representative ISF measurement for the clean Ag(001) surface, where the solenoid current was varied from −1 to +1 A, with 1025 equally spaced points. The upper panel displays the real and imaginary components of the polarisation as a function of solenoid current. The lower panel presents the corresponding energy spectrum, obtained by Fourier transformation and conversion from wavelength to energy.^42,74,75^ Distinct loss and gain features reflect the creation and annihilation of surface phonons, yielding spectra analogous to time-of-flight (TOF) measurements.

## 3 Results and discussion

### 3.1 Surface phonon dispersion

Before considering linewidths, lifetimes, or anharmonic effects, it is essential to first establish the surface phonon dispersion itself. In the acoustic energy range, two experimental approaches have traditionally been used to determine dispersions of crystalline surfaces: high-resolution electron energy loss spectroscopy (HREELS) and helium atom scattering (HAS). While HREELS is particularly effective at higher phonon energies, classical HAS offers superior energy resolution for the very low-energy acoustic modes.^30^ The present HeSE measurements build on this capability, providing access not only to the dispersion but also to the intrinsic linewidths.

Surface phonon energies were obtained by performing spin-echo measurements over a range of spin-echo times, with Fig. 1(b) showing a typical measurement. Oscillations in the measured polarisation correspond to a lattice vibration with a characteristic period, *i.e.*, to a particular surface phonon mode. The corresponding inelastic spectrum is obtained *via* Fourier transforming the data to the wavelength domain and converting to the energy scale, with energy loss and gain peaks for the creation and annihilation of a phonon, respectively^42,75^ (lower panel in Fig. 1(b)). The phonon energy *E*_ph_ = *ħω* is then given *via*:2
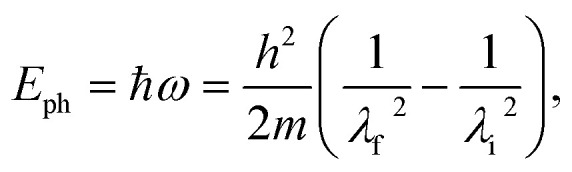
where *λ*_i_ and *λ*_f_ are the incoming and outgoing wavelengths of the He beam, and *m* is the ^3^He mass.

To determine the entire phonon dispersion curve up to the Brillouin zone boundary, a series of spin-echo spectra at varying incident angles *ϑ*_i_ was measured. The phonon dispersion was then obtained by calculating the parallel momentum transfer |*Q*| for each extracted phonon energy Δ*E* based on the conservation laws of energy and parallel momentum, which provide the so-called scancurve for planar scattering:^42,75^3
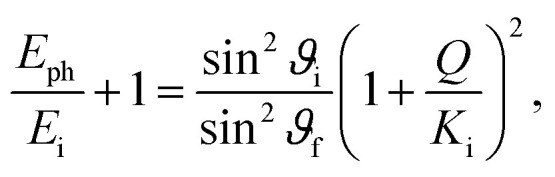
where *E*_i_ is the energy of the incident beam and *K*_i_ is the parallel component of the incident wavevector. Here, *ϑ*_i_ and *ϑ*_f_ are the incident and final angles with respect to the surface normal, respectively. The phonon parallel momentum is then given by |*Q*| + *G*, with the surface reciprocal lattice vector *G* in the scattering plane, needed to bring *Q* into the first Brillouin zone, accounting for *umklapp* processes.

**Fig. 2:**
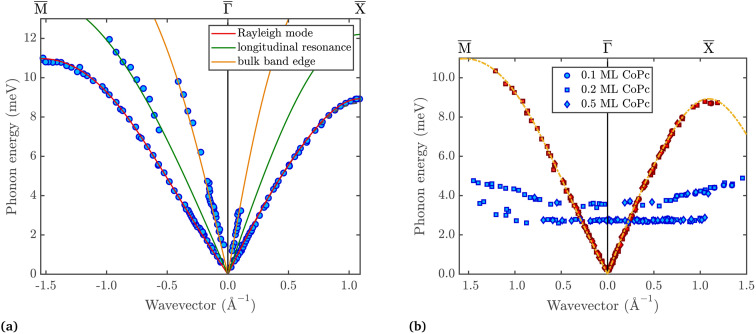
Measured phonon dispersion relation for the pristine Ag(001) surface and for the CoPc-covered surface. (a) The extracted phonon dispersion of Ag(001) in the energy region of acoustic phonons for the two high-symmetry directions with the sample held at room temperature (blue symbols). Solid lines represent simple sinusoidal fits to the observed modes. The Rayleigh mode exhibits the highest intensity across the Brillouin zone. Additionally, the longitudinal resonance and the bulk band edge are clearly discernible. (b) Extracted phonon dispersion for the CoPc-covered Ag(001) surface. The Rayleigh mode of the underlying Ag(001) surface is still clearly visible and plotted as red symbols, while blue symbols are assigned to new vibrational modes due to the adsorbed CoPc molecules. Here, different symbols correspond to different coverages of CoPc in monolayer (ML).

Fig. 2(a) shows the extracted phonon dispersion of Ag(001) in the acoustic energy range for the two principal high-symmetry directions, 
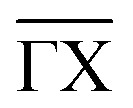
 and 
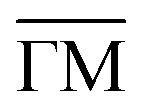
, with all measurements performed at room temperature. Three distinct features are observed consistently across the dispersion curves. The lowest-lying branch corresponds to the Rayleigh wave (RW), the fundamental acoustic surface mode, which exhibits the highest intensity throughout the Brillouin zone due to its surface-localised character.^75^ Above the RW, the longitudinal resonance (LR) appears; however, close to Γ̄ this mode is difficult to resolve because it manifests in the spectra mainly as a weak shoulder adjacent to the much stronger RW peak. A third discernible feature is attributed to the bulk band edge, which generates a distinct peak across the measured wavevector range. Notably, only very few experimental data for this bulk-related feature exist in the literature.^76^ Upon comparison with the RW mode measured previously by HREELS,^31^ the RW energies extracted in Fig. 2(a) appear slightly lower when approaching the Brillouin-zone boundary, a deviation that may arise from subtle differences in probing depth, scattering geometry, or experimental resolution.

For comparison, Fig. 2(b) displays the measured dispersion for the CoPc-covered Ag(001) surface. In addition to the underlying Ag(001) RW, which remains clearly visible (red symbols), adsorption of CoPc gives rise to two new vibrational modes. These modes show hardly any dispersion across the measured wavevector range, consistent with frustrated molecular vibrations that are highly localised and only weakly coupled to the substrate lattice.^77,78^ A detailed discussion of these adsorbate-induced modes, including their assignment and dynamical implications, will be presented in a later section.

**Fig. 3:**
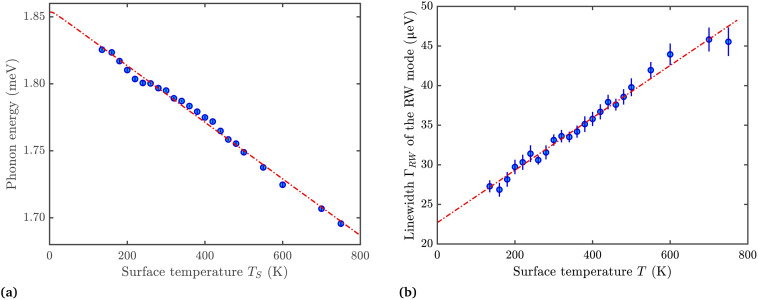
Temperature-dependent properties of the Rayleigh wave (RW) mode on Ag(001). (a) Temperature dependence of the phonon energy *E*_ph_ for the RW mode at a wavevector *Q* = 0.15 Å^−1^ along 
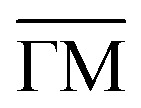
. In a first approximation, the mode softening with increasing temperature can be fitted according to (4) (red dash-dotted line). (b) Intrinsic linewidth *Γ*_RW_ of the RW phonon mode at *Q* = 0.15 Å^−1^*versus* surface temperature. A linear fit (red dash-dotted line) is applied to the data, with extrapolation to *T* = 0 K enabling determination of the intrinsic linewidth and corresponding phonon lifetime at zero temperature.

The position and linewidth of the RW mode were then measured between 135 K and 750 K along the 
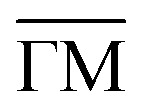
 azimuth as shown in Fig. 3. For an accurate determination of both the phonon energy and the linewidth, each spectrum in the energy-loss domain, obtained after Fourier transforming the measured signal, was fitted using the procedure illustrated in the inset of Fig. 1(b). This provides a consistent and precise extraction of the peak position and full width at half maximum (FWHM) for the RW mode at each temperature (see also Fig. S1(b) in the SI).

Before determining the intrinsic linewidth of the RW mode, it is necessary to consider the experimental factors that may contribute to the observed broadening. The diffuse elastic peak at Δ*E* = 0, which occurs due to the presence of defects such as steps, does not affect the present analysis, since our linewidths are extracted from finite-energy phonon peaks. In “classical” HAS, the energy resolution of the apparatus will add to the width of the peak; however, in the spin-echo setup, tuned for measurements of the quasi-elastic peak (around Δ*E* = 0), the measurement is only limited by the maximum Fourier time of the instrument. On the one hand, upon using the quasi-elastic setup for phonon measurements, an additional broadening may arise from the projection of the inelastic signal through the wavelength-intensity transfer matrix.^74^ On the other hand, this projection effect can be removed by selecting the correct tilt angle following measurements of the wavelength intensity function *ρ*(*λ*_i_,*λ*_f_), as described in ref. 42 and 74, as well as the SI. As shown in Fig. S1(a) of the SI, it allows the determination of the optimum tilt angle (eqn (S5) in the SI) for resolving the intrinsic linewidth. For the RW creation peak, it is found to be *α* = 140.77°, approximately +6° from the quasi-elastic condition at *α* = 135°. Once this tilt correction is applied, the only remaining experimental broadening arises from the angular resolution: specifically, the finite angular spread of the incident beam causes a small broadening where the scan curve intersects the phonon dispersion. This contribution is accounted for explicitly in the linewidth analysis presented later. These measurements form the basis for determining both the anharmonicity of the RW energy shift and the intrinsic lifetime derived from the linewidth analysis presented in the following sections.

### 3.2 Temperature dependence of the phonon energy

The temperature dependence of surface phonon energies provides a sensitive probe of anharmonicity, reflecting deviations from the ideal harmonic lattice behaviour that arise from the intrinsic asymmetry of the surface potential. At a crystal surface, the reduced coordination of atoms leads to an anharmonic restoring force that includes, in lowest order, a cubic term. This anharmonicity manifests experimentally as a softening of phonon frequencies and an increase in linewidths with increasing temperature. Such behaviour has been reported for clean metal surfaces including Al(001), Al(111), Cu(001) and Cu(110), using both HAS and HREELS.^27,29–31,79,80^ Quantitatively, the temperature-induced softening is commonly described using an expression adapted from the theory of molecular vibrations, in which the decrease of the phonon energy is proportional to the harmonic energy and to the thermal occupation number.

The temperature-dependent position of the RW is plotted in Fig. 3(a). The weak monotonic softening observed between 135 K and 750 K is consistent with the general behaviour of surface modes, though notably smaller in magnitude than for the higher-energy modes typically examined in earlier studies. The slope of 210 neV K^−1^ and the extrapolated harmonic energy of 1.86 meV at *T* = 0 indicate an exceptionally slight anharmonic response. To quantify this behaviour, the experimental data were fitted using the standard form

4 *ħω*(*T*) = *ħω*_0_ − *χ*_e_*ħω*_0_(2*n*_0_ + 1),

where *χ*_e_ is the anharmonicity parameter and the occupation of the mode *n*_0_ is *n*_0_ = exp(*ħω*_0_/*k*_B_*T*) − 1. The resulting anharmonicity constant,*χ*_e_ = (1.23 ± 0.02) × 10^−3^is approximately an order of magnitude smaller than the values reported for clean metal surfaces, for which *χ*_e,s_ ≈ 0.014–0.024.^30^ However, all systems discussed in ref. 30 were measured at the Brillouin-zone boundary, where phonon energies are significantly higher and anharmonic effects correspondingly more pronounced. In contrast, the present measurement concerns a long-wavelength RW mode of very low energy (1.86 meV at *T* = 0) at a small wavevector of *Q* = 0.15 Å^−1^. The wavevector dependence of surface phonon anharmonicity is not known in detail, and, to the best of our knowledge, no temperature-dependent measurements exist for phonon modes at such low energies and small wavevectors. Consequently, the comparatively small anharmonicity observed here should be interpreted as a characteristic feature of this long-wavelength, low-energy mode rather than an anomaly relative to the higher-energy zone-boundary phonons discussed in previous work.

It should be mentioned that the temperature dependence of the phonon energy itself contains information about the real part of the phonon self-energy, reflecting contributions from anharmonicity and e–ph renormalisation, which is further discussed below. A quantitative analysis typically involves comparing the experimentally determined *ω*(*T*) with anharmonic lattice-dynamics calculations and, where available, first-principles e–ph calculations.^81^ Such comparisons allow the respective contributions to the renormalisation of the Rayleigh-mode dispersion to be disentangled, and would provide a further avenue for benchmarking theory against the present experimental results.

### 3.3 Linewidth and lifetime of the Rayleigh wave

We now turn to the linewidth and lifetime of the RW mode. The temperature dependence of the RW mode’s linewidth is extracted from fits to the spectra as shown in Fig. S1(b) of the SI. As discussed above, several resolution effects can contribute to the measured linewidth of a phonon mode and must be disentangled from the measured broadening. The energy resolution of the incident beam is addressed directly by selecting an appropriate tilt angle during measurement (*α* = 140.77°, see Fig. S1(a) in the SI). Any remaining contribution arises from angular broadening within the experimental apparatus. This is estimated based on the FWHM of the specular peak, measured as 0.088° (see Fig. S2(a) in the SI). Using this angular width and the phonon dispersion relation from the scan curve (3), we obtain an apparatus-induced energy broadening of Δ*E*_app_ = 30 µeV. This broadening adds to the linewidth broadening as *Γ*_app_ and is computed for each phonon measurement by numerically solving the scan curve equation. The intrinsic or “natural” linewidth of the RW mode is then given by (*Γ*_RW_)^2^ = (*Γ*_total_)^2^ − (*Γ*_app_)^2^, where *Γ*_total_ is the total measured linewidth. Following this procedure, the intrinsic linewidth *Γ*_RW_ of the RW mode at *Q* = 0.15 Å^−1^ is obtained, and its temperature dependence is shown in Fig. 3(b).

The extracted intrinsic linewidths *Γ*_RW_ of the Rayleigh wave lie in the range of approximately 25–45 µeV across the measured temperature regime. Using the standard relation between linewidth and lifetime,5
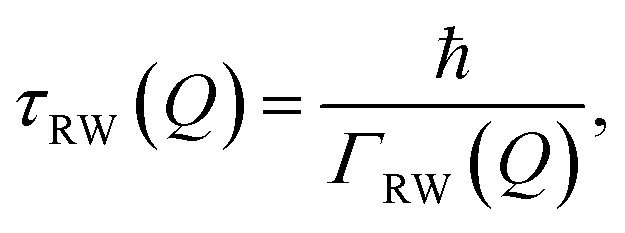
the intercept of the linear fit in Fig. 3(b) can be used to extrapolate the lifetime to *T* = 0 K, yielding*τ*_RW_ = (29 ± 1) ps.

At low temperatures, the lifetime of a surface phonon mode such as the Rayleigh wave on Ag(001) is governed by temperature-independent scattering channels. In the limit *T* → 0, anharmonic phonon–phonon interactions vanish, as they require a thermally populated phonon bath. The remaining contributions to the phonon linewidth *Γ*(*Q*) arise from elastic or quasi-elastic processes, most notably defect and disorder scattering, as well as e–ph coupling. In a noble metal such as silver, with a well-defined Fermi surface, the Rayleigh mode at finite parallel wavevector *Q* can decay into electron–hole pairs provided both energy and momentum conservation are satisfied. This process results in a finite e–ph self-energy, and hence a non-zero linewidth even at *T* = 0. Additional temperature-independent contributions arise from isotopic disorder and residual surface imperfections, such as steps, terraces, or dislocations.^48^

In general, bulk phonon modes exhibit longer intrinsic lifetimes due to their propagation through the material interior, where surface scattering is minimal and environmental coupling is relatively weak, particularly in high-purity crystals and at low temperatures for acoustic modes. Surface phonon modes, on the other hand, are localised at surfaces or interfaces and are more strongly coupled to their environment, including surface defects, adsorbates, and other excitations such as electronic or photonic modes in nanostructures. This enhanced coupling, together with additional scattering arising from confinement and morphological irregularities, leads to stronger damping and hence shorter lifetimes. Moreover, first-principles calculations of phonon lifetimes in simple metals have demonstrated that low-temperature lifetimes are strongly dependent on the phonon branch and wavevector, with long-wavelength acoustic modes typically exhibiting extended lifetimes due to reduced scattering phase space and weaker coupling to electronic excitations.^81^ In this context, the experimentally observed Rayleigh-mode lifetime of *τ* ≈ 29 ps at *Q* ≈ 0.15 Å^−1^ reflects the combined influence of e–ph interactions and residual elastic scattering from static disorder at the surface.

A comparison with bulk phonon transport further places this value into perspective. In crystalline solids, mean free paths vary strongly with phonon branch and frequency: for example, ref. 10 reports bulk mean free paths at 300 K ranging from approximately 120 to 20 nm depending on phonon wavevector and energy. Similarly, time-resolved measurements in PbTe show bulk transverse acoustic lifetimes of order 10 ps at phonon energies around 4 meV at 300 K.^82^ As mentioned above, surface modes generally experience stronger environmental coupling and thus shorter lifetimes than their bulk counterparts; however, the Rayleigh-wave lifetime reported here is of comparable magnitude to these bulk values, underscoring that surface-specific damping channels, though significant, do not necessarily lead to dramatically shorter lifetimes at the measured wavevector and temperature range. In this context it should also be mentioned that acoustic phonons typically possess much longer lifetimes than optical phonons, owing to their lower energies and correspondingly limited decay phase space. By contrast, optical phonons decay more readily into multiple lower-energy excitations and therefore exhibit shorter intrinsic lifetimes.

Before turning to finite-temperature scattering mechanisms, we briefly consider the spatial implications of the extracted lifetime. The finite phonon lifetime can be translated into a characteristic propagation length along the surface. A simple estimate is given by6
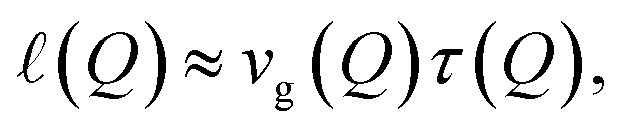
where *v*_g_(*Q*) is the group velocity of the Rayleigh wave at the measured momentum transfer. From the slope of the dispersion in Fig. 2(a), we estimate *v*_g_ ≈ 1.5 × 10^3^ m s^−1^. Inserting the experimentally determined lifetime *τ* ≈ 29 ps into eqn (6) yields a propagation length of
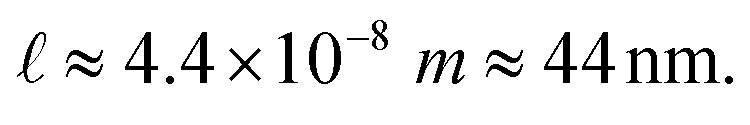


This length scale characterises the distance over which the Rayleigh mode can propagate coherently before decaying and is particularly relevant for discussions of nanoscale energy transport and surface-phonon-mediated coupling phenomena.

Returning to the temperature-dependence of the linewidth, we note that from an approximately linear increase of *Γ*(*T*) with temperature, the slope can be associated with thermally activated scattering processes. In the temperature regime where anharmonic three-phonon processes are weak for the surface mode in question, the dominant temperature-dependent contribution is expected to arise from e–ph scattering. Under this assumption, one may write

7 *Γ*(*T*) ≈ *Γ*_0_ + *Γ*_e–ph_(*T*),

where *Γ*_0_ is the residual (temperature-independent) contribution from defects, disorder, and the *T* = 0 part of e–ph coupling, while *Γ*_e–ph_(*T*) carries the leading temperature dependence. The slope d*Γ*/d*T* then provides a direct measure of the strength of the e–ph interaction for this particular Rayleigh mode. In a simple linear-in-*T* model for the e–ph contribution,

8 *Γ*_e–ph_(*T*) ≃ 2π*λk*_B_*T*,

one obtains9
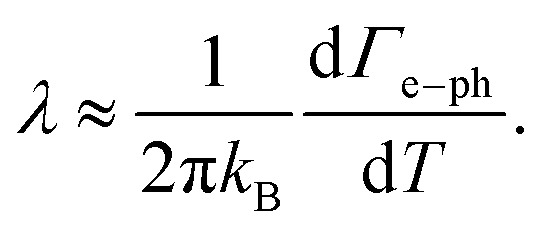


From the experimentally determined slope of the linewidth in Fig. 3(b), d*Γ*/d*T* = (33 ± 1) neV K^−1^, *i.e.*, (3.32 ± 0.10) × 10^−8^ eV K^−1^, together with the Boltzmann constant *k*_B_, we obtain a mode-resolved e–ph coupling constant of*λ*_RW_ = (6.1 ± 0.2) × 10^−5^indicating an extremely weak e–ph interaction for this particular surface Rayleigh phonon. However, this value is strongly mode- and wavevector-specific and should not be compared directly to averaged *λ* parameters, which integrate contributions from all phonon branches.^30,45^

It is interesting to compare the temperature dependence and intrinsic linewidths obtained here with those recently reported for Ru(0001), where HeSE measurements revealed substantially stronger damping and broader linewidths of the Rayleigh mode.^14^ Compared to Ag(001), the linewidth on Ru(0001) reflects sizeable and strongly temperature-dependent contributions from e–ph, phonon–phonon, and defect-induced scattering. Notably, the temperature dependence is non-monotonic: the linewidth initially decreases with increasing temperature before rising again at higher temperatures, a behaviour attributed to dominant e–ph scattering at low and intermediate temperatures and to anharmonic phonon–phonon interactions only becoming significant above several hundred kelvin. This combination of strong e–ph coupling and defect scattering produces linewidths on Ru(0001) that are significantly broader than those measured here for Ag(001).

As shown in Fig. S2(b) in the SI, small but systematic deviations from strictly linear behaviour are also observed for both the phonon energy and the linewidth of the RW mode in the present case. For the phonon energy, this suggests that a simple anharmonicity model may not fully account for all temperature-dependent contributions. For the linewidth, although e–ph interactions may display a temperature dependence as described above,^14^ the effect appears comparatively weak in Ag(001). Moreover, phonon–phonon interactions, which often produce an approximately linear broadening with temperature, depend sensitively on the available decay channels and on the coupling to multiphonon processes. In the present system these deviations remain minor, underscoring the suitability of Ag(001) as a model surface for phonon-lifetime studies, while simultaneously motivating more detailed microscopic modelling in future work.

Before extending the discussion to additional adsorbate-induced vibrations, it is useful to briefly revisit the theoretical landscape. From a theoretical perspective, quantitative predictions of phonon linewidths as a function of wavevector and temperature remain challenging, particularly for surface modes. Early studies, performed at the Brillouin-zone boundary, already demonstrated the sensitivity of surface-phonon damping to e–ph coupling and anharmonicity, but did not access its wavevector dependence.^83^ Modern first-principles approaches still face a fundamental limitation: accurate linewidth calculations at specific momenta require large supercells, rendering systematic wavevector-resolved studies computationally demanding. Machine-learning-accelerated interatomic potentials offer a promising route to overcome these constraints and enable broader, high-resolution surveys of surface phonon lifetimes.^84,85^ Progress in this direction would enable the establishment of systematic trends across elements and surface orientations, as suggested by recent bulk-focused studies,^86^ and would clarify to what extent surface truncation, *e.g.* comparing (111) and (001) facets, modifies the available decay channels and alters the balance between e–ph and phonon–phonon interactions. In this context, surface phonon linewidths and their e–ph contributions are of particular interest for quantum and low-dimensional materials where e–ph coupling plays a key role, including graphene,^61,87^ topological insulators,^46,88,89^ and other systems where surface states or Dirac-like dispersions enhance quasiparticle–phonon interactions. Understanding phonon damping in such materials is essential not only for describing energy dissipation, but also for predicting macroscopic properties such as thermal expansion, flexural rigidity, and the mechanical stability of two-dimensional materials.^90,91^ Establishing a unified microscopic framework capable of treating both conventional metal surfaces and quantum materials on equal footing would therefore represent an important advance.

### 3.4 Vibrational modes and lifetimes of adsorbed molecules

Finally, we turn to adsorbate vibrations and their lifetime or vibrational damping. While Raman scattering is frequently used to obtain chemical information on adsorbed molecules, providing access to internal molecular vibrations and symmetry assignments,^92^ it does not deliver wavevector-resolved information and therefore cannot probe the dispersion of adsorbate modes or their lateral coupling. Instead, high-resolution HAS is uniquely capable of resolving adsorbate vibrations in the low-energy regime (<20 meV), particularly the parallel frustrated-translation (T) modes and perpendicular frustrated-stretch (S) modes that are often inaccessible to infrared or electron energy-loss techniques due to their weak dipole activity and typically small dynamic dipole moments.^77^ The vibrational dynamics of adsorbates on metal surfaces provide essential insight into the shape and anisotropy of the adsorption potential as well as the coupling between adsorbates and substrate phonons. Frustrated translations and frustrated rotations probe the curvature of the adsorption well parallel and perpendicular to the surface, offering a quantitative window into molecule-surface bonding and lateral interactions.^30,77,78^ HAS measurements have revealed systematic trends in adsorbate vibrational energies across physisorbed and chemisorbed systems, clarified the role of adsorption geometry, and enabled the identification of collective adsorbate phonon branches, including dispersion arising from intermolecular interactions.^77^ Beyond characterising molecule–surface binding, adsorbate vibrational frequencies serve as benchmarks for theoretical treatments of adsorbate–substrate interactions.^93^

Large organic adsorbates on metal surfaces exhibit a rich spectrum of dynamical processes, ranging from long-range diffusion to local vibrational motion, each of which influences molecular assembly, reactivity, and functionality.^70,94^ CoPc provides a prototypical case, combining substantial molecular mass with a planar macrocyclic structure and a central metal atom, features that give rise to characteristic adsorbate–substrate interactions. Recent HeSE diffusion studies on CoPc/Ag(001) have resolved its lateral mobility and adsorption landscape.^52,68^ Here, we extend this picture by probing the low-energy vibrational modes of CoPc using HeSE, thereby accessing the frustrated translations and other adsorbate-specific excitations that govern the local curvature of the adsorption potential and the coupling to substrate phonons.

**Fig. 4:**
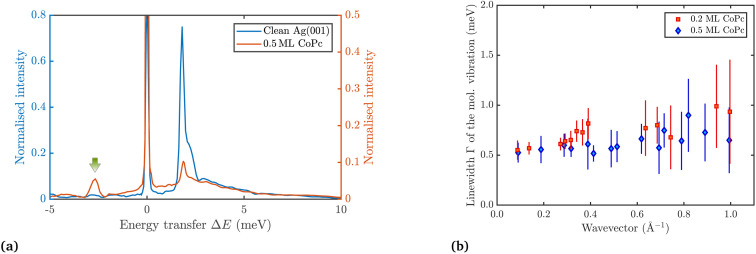
(a) Comparison of an exemplary phonon spectrum for CoPc/Ag(001) and pristine Ag(001) under the same kinematic experimental conditions. (b) Extracted linewidths for the frustrated translation of CoPc here shown for the molecular vibrations at *E*_0_ ≈ 2.77 meV along the 
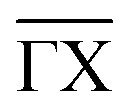
 direction.

Therefore, HeSE measurements were subsequently performed after depositing CoPc on Ag(001) to a submonolayer coverage of 0.1–0.5 ML, with coverage calibration carried out following the procedure described in ref. 68. A representative energy-domain spectrum is shown in Fig. 4(a), together with a corresponding spectrum of pristine Ag(001) recorded under the same kinematic conditions. Upon CoPc adsorption, the Rayleigh mode remains discernible but exhibits much less intensity, and an additional inelastic feature appears at an energy of approximately 2.75 meV, characteristic for a vibrational mode from molecular adsorbates. The full phonon dispersion extracted for the CoPc/Ag(001) system is presented in Fig. 2(b). Within the sampled coverage range, no discernible difference in the dispersion is observed for the two distinct low-energy modes, with characteristic energies of *E*_0_ ≈ 2.77 and 3.5 meV upon reaching the surface Brillouin zone centre Γ̄. While some wavevector dependence is observed, the dispersion of the molecular mode is comparatively weak, as discussed further below. These features reflect the vibrational response of the adsorbed CoPc molecules and form the basis for the subsequent analysis of mode character, dispersion, and coupling to the substrate.

We start by discussing the two distinct low-energy branches in Fig. 2(b) as induced upon CoPc adsorption. A single frustrated-translation (T) mode would be expected if all molecules occupied an equivalent adsorption configuration; however, CoPc on Ag(001) adopts two preferred rotational orientations with respect to the substrate lattice, corresponding to locally similar but not symmetry-equivalent adsorption geometries. Because the frustrated translations probe the curvature of the adsorption well associated with each orientation, the two configurations yield slightly different harmonic restoring forces, producing the distinct zone-centre energies of *E*_0_ ≈ 2.77 and 3.5 meV. Both branches display an approximately parabolic dispersion with parallel wavevector *Q*, indicative of weak, long-range repulsive interactions between adsorbates in the dilute regime. Such interactions lead to a quadratic increase of vibrational energy with *Q*, consistent with the trends previously inferred from CoPc diffusion on Ag(001).^68^ The lower-energy branch exhibits only minimal *Q*-dependence, signalling weaker intermolecular coupling for that rotational configuration, whereas the higher-energy branch shows a more pronounced curvature due to stronger effective lateral repulsion. This behaviour parallels observations for other planar organic adsorbates, such as cyclopentadienyl on Cu(111), where HeSE measurements revealed two non-dispersive frustrated modes.^78^

The linewidths extracted for the lower-energy CoPc vibrational mode along 
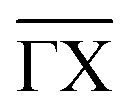
 are shown in Fig. 4(b). The molecular mode exhibits a substantial broadening of the order *Γ* ≈ 0.5 meV at low *Q*, increasing to values approaching 1 meV at larger wavevector *Q*. Such linewidths are considerably broader than those typically observed for small chemisorbed molecules, reflecting the stronger damping expected for a large, multi-ring adsorbate with many internal degrees of freedom and a sizeable adsorbate–substrate contact area. It is important to note that the present measurements were conducted at *T* = 300 K and were not optimised with respect to the instrumental tilt angle, implying that additional resolution contributions may add to the intrinsic linewidth. A modest variation of *Γ*(*Q*) is observed, particularly as the wavevector approaches the de Gennes narrowing condition associated with enhanced in-plane correlations.^68^ While a slight difference between the two sampled coverages (0.2 and 0.5 ML) cannot be excluded, any such trend remains within the experimental uncertainties.

The overall magnitude and *Q*-dependence of the CoPc linewidths may be contrasted with those reported for smaller adsorbates. For cyclopentadienyl (Cp) on Cu(111), Lechner *et al.* observed vibrational dephasing widths of several meV that increase linearly with wavevector *Q* and exhibit clear signatures of coupling to the substrate phonon bath, with an intercept corresponding to a lifetime of only a few picoseconds.^78^ In the present CoPc system, the broadening is smaller in absolute terms but comparable when scaled by the molecular mass, and the more moderate *Q*-dependence likely reflects the weaker lateral coupling and larger inertia of CoPc relative to Cp. By contrast, for CO adsorbed on metal surfaces such as Cu(001), vibrational linewidths associated with frustrated translations are typically narrower, as demonstrated in QHAS studies,^77,95^ since the intrinsic damping is reduced due to the small mass and more localised adsorption geometry of the CO molecule. This comparison is consistent with expectations: larger, more complex adsorbates with extended contact areas, such as CoPc, couple more strongly to the substrate and thus display enhanced vibrational damping relative to compact species like CO.

While the present measurements already reveal clear signatures of vibrational damping and intermolecular coupling, a more complete picture will require future HeSE experiments with optimised instrumental resolution, extended *Q* ranges, and temperature-dependent studies to disentangle different contributions to both the linewidths and mode energies of molecular vibrations. Nonetheless, these results establish HeSE as a sensitive probe of vibrational motion in large adsorbates and offer new insight into the coupling between molecular vibrations and substrate phonons for CoPc on Ag(001).

## 4 Conclusions

In this work, we have demonstrated that helium spin-echo spectroscopy provides direct access to the intrinsic linewidths and lifetimes of low-energy surface phonons and adsorbate vibrations with sub-µeV sensitivity. For Ag(001), we established the full dispersion of the Rayleigh wave, quantified its exceptionally small anharmonicity, and extracted an intrinsic linewidth of only 25–45 µeV across the measured temperature range. The resulting lifetime of *τ*_RW_ ≈ 29 ps and propagation length of 
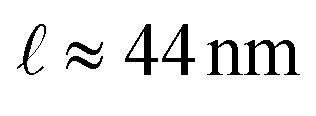
 demonstrate that long-wavelength surface phonons can retain coherence over surprisingly large distances even in the presence of e–ph and defect-induced scattering. The extracted e–ph coupling constant, *λ*_RW_ ≈ 10^−4^, confirms that long-wavelength acoustic modes at Ag surfaces may couple only very weakly to the electronic system, in stark contrast to the substantially stronger damping observed on Ru(0001), where much larger contributions from e–ph and anharmonic processes dominate the linewidth.

Extending the method to CoPc/Ag(001), we resolved two frustrated-translation modes arising from distinct rotational adsorption geometries and characterised their weak dispersion, and vibrational damping. The observed linewidths of *Γ* ≈ 0.5–1 meV reflect the complexity and enhanced dissipation channels associated with large organic adsorbates, in agreement with trends seen for other molecular systems. These results establish HeSE as a sensitive probe of low-energy vibrational dynamics in hybrid organic–metal interfaces and open avenues for quantifying vibrational lifetimes, intermolecular interactions, and potential-energy landscapes of large adsorbates.

Looking ahead, several directions emerge from the present work. Methodologically, further improvements in instrumental resolution and systematic temperature-dependent studies will refine the separation of e–ph, phonon–phonon, and defect-driven damping mechanisms. On the theoretical side, quantitative predictions of wavevector-resolved phonon linewidths remain computationally demanding due to the need for large supercells; nonetheless, machine-learning-accelerated approaches offer a promising route to enable broader and more efficient modelling. More broadly, extending such combined experimental and theoretical methodologies across different elements, surface orientations, and reduced-dimensional systems will help establish general trends in vibrational lifetimes and surface energy dissipation. This is particularly relevant for materials in which phonons strongly influence transport and quasiparticle behaviour, including 2D materials, topological systems, correlated oxides, and other emerging quantum materials.

In summary, the present results demonstrate that HeSE provides a powerful and versatile approach for probing vibrational lifetimes at surfaces, from long-wavelength acoustic phonons to frustrated molecular modes. By coupling high-resolution surface-sensitive experiments with advancing theoretical tools, a comprehensive microscopic framework for phonon-mediated energy dissipation at surfaces is within reach, with implications for catalysis, thermal transport, molecular functionality, and the behaviour of quantum materials.

## Conflicts of interest

There are no conflicts to declare.

## Supplementary Material

FD-265-D5FD00162E-s001

## Data Availability

The data supporting the findings of this study are available on https://repository.tugraz.at/ with the DOI https://doi.org/10.3217/1r9j9-4vj12. Supplementary information (SI): additional details on the HeSE measurements and on the experimental setup required for measuring the intrinsic linewidth. See DOI: https://doi.org/10.1039/d5fd00162e.
