# Supplementary material for: Helium spin-echo as a surface-sensitive probe of vibrational energy dissipation
Source: Faraday Discuss. 2026 Jun 8. Online ahead of print. doi: 10.1039/d5fd00162e (PMC13244377; doi:10.1039/d5fd00162e)
Supplement: FD-OLF-D5FD00162E-s001 [file FD-OLF-D5FD00162E-s001.pdf]

Supplementary Information (SI) for Faraday Discussions.
This journal is © The Royal Society of Chemistry 2026

Supplementary Information: Helium Spin-Echo as a Surface-Sensitive Probe of Vibrational Energy Dissipation

Experimental Details

As described in the main text, HeSE measures the ISF, i.e. the polarisation of the ^{3}He beam after surface scattering following controlled manipulation of the nuclear spin via the magnetic fields generated by currents through the incoming and outgoing solenoids. With I_i and I_f denoting the currents in the respective solenoids, the associated magnetic field may be written as $B_{\mathrm{eff}} = \int_0^L \frac{B(z)}{I}\,\mathrm{d}z$, which defines a coil-specific constant, the coil sensitivity.
The interaction time of the ^{3}He atoms with the solenoid field is proportional to their wavelength λ. Consequently, the spin-echo lineshape corresponds to the Fourier transform of the scattering probability in the wavelength domain, $\rho(\lambda_i \rightarrow \lambda_f)$.[R1,R2]
If the spin polarisation is rotated in the xy-plane and initially aligned along the x-axis, the final polarisation detected after scattering is given by[R1,R3]

$$P_x(I_i, I_f) = \int \rho(\lambda_i, \lambda_f) \cos\left[\frac{\gamma m B_{\mathrm{eff}}}{h}(I_i\lambda_i + I_f\lambda_f)\right] \mathrm{d}\lambda_i\,\mathrm{d}\lambda_f, \tag{S1}$$

where γ is the gyromagnetic ratio of ^{3}He. The measured polarisation P_x thus depends on the solenoid currents (I_i, I_f) and on the distribution of initial and final wavelengths. The wavelength intensity function $\rho(\lambda_i, \lambda_f)$ specifies the probability that an atom with initial wavelength λ_i scatters into a final wavelength λ_f and subsequently reaches the detector.
To simplify the description of the experiment, it is convenient to introduce the parameter κ, which is directly proportional to the applied solenoid current[R1,R2,R4]:

$$\kappa \equiv \frac{1}{2\pi h}\gamma m B_{\mathrm{eff}} I. \tag{S2}$$

The measured complex polarisation $P(\kappa_i, \kappa_f)$ is therefore a function of the experimentally tunable quantities κ_i and κ_f. The spin-echo process can then be expressed as a two-dimensional Fourier transform:[R1,R3,R4]

$$P(\kappa_i, \kappa_f) = \int \rho(\lambda_i, \lambda_f)\, \mathrm{e}^{2\pi\mathrm{i}(\kappa_i\lambda_i + \kappa_f\lambda_f)}\, \mathrm{d}\lambda_i\,\mathrm{d}\lambda_f, \tag{S3}$$

where $\rho(\lambda_i, \lambda_f)$ is the wavelength intensity matrix, defining the probability distribution of scattered atoms in the two-dimensional wavelength space. Because of the Nyquist and resolution constraints, reconstructing $\rho(\lambda_i, \lambda_f)$ via the full inverse Fourier transform typically requires long acquisition times. An efficient reconstruction strategy based on image-reconstruction techniques is described in Ref.[R1]. We employ this approach here to obtain $\rho(\lambda_i, \lambda_f)$ for Ag(001), shown in Figure S1(a), and from it determine the corresponding optimum tilt angle as discussed below.
Since the wavelength intensity is concentrated around the mean initial wavelength, it may also be regarded as a one-dimensional function broadened into two dimensions by the spread of incident wavelengths. When only quasi-elastic broadening is of interest, it is therefore common to measure along a single line in (κ_i, κ_f) space. In practice, this corresponds to maintaining a constant ratio κ_f/κ_i, i.e. a fixed ratio of solenoid currents[R1,R2]. According to the Fourier slice theorem[R1], a measurement along such a line yields the Fourier transform of the projection of the full $\rho(\lambda_i, \lambda_f)$ matrix onto the corresponding line in wavelength space. For a projection defined by the angle α, Eq. (S3) reduces to the 1D Fourier transform

$$P_\alpha(\kappa) = \int \rho_\alpha(\lambda)\, \mathrm{e}^{2\pi\mathrm{i}\kappa\lambda}\, \mathrm{d}\lambda, \tag{S4}$$

with $\rho_\alpha(\lambda)$ the projected wavelength intensity function.
In principle, measurements may be performed along any projection angle α, which can be chosen to optimise the resolution of a particular spectral feature. The curvature of scattering events in $\rho(\lambda_i, \lambda_f)$ implies that no single projection is ideal for all features[R1,R2]. A quasi-elastic measurement yields the true quasi-elastic linewidth but overestimates the linewidth of inelastic modes. The optimum angle α for a specific inelastic scattering event follows from[R4]

$$\frac{\mathrm{d}\lambda_f}{\mathrm{d}\lambda_i} = -\tan\alpha = -\frac{I_i}{I_f}, \tag{S5}$$

a relation of particular importance when determining the intrinsic linewidth, and therefore the lifetime of a phonon mode.

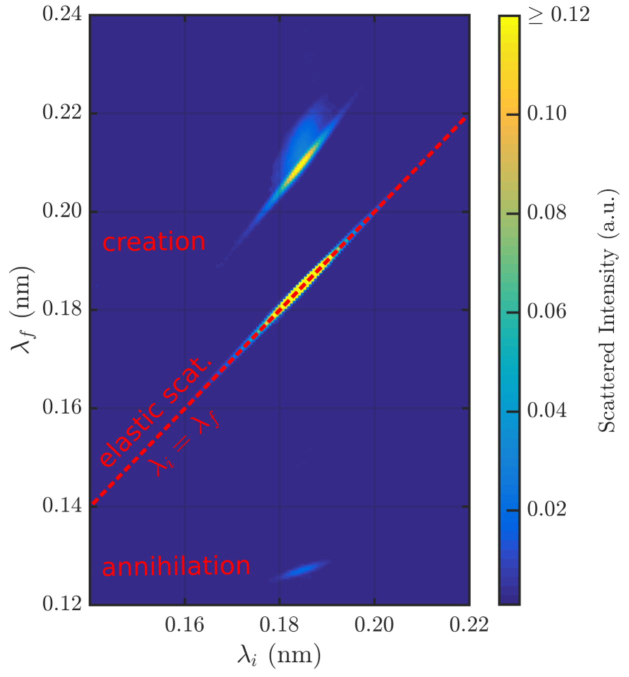

(a) Measurement of the wavelength intensity function $I(\lambda_i, \lambda_f)$ at an incident angle $\vartheta_i = 19.2°$, performed at $T = 120$ K, used to determine the optimum tilt angle for resolving the linewidth of the inelastic creation event. The resulting optimum projection angle is $\alpha = 140.77°$, differing by approximately $+6°$ from the elastic optimum of $\alpha = 135°$.

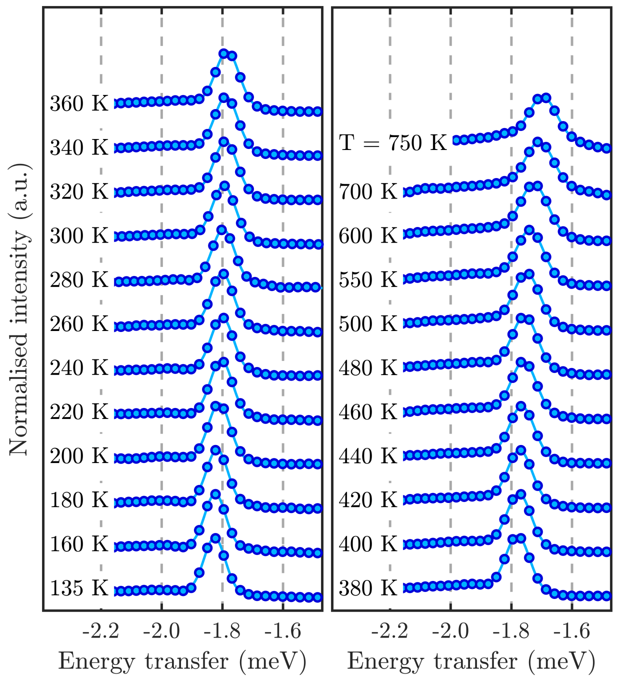

(b) Temperature dependence of the measured spectra around the position of the RW creation event.

Figure S1 (a) and (b) present complementary data sets pertaining to the inelastic RW creation process, examining the optimal angular projection and its thermal variation.

Once the optimum projection angle α has been identified for a specific phonon excitation, here the Rayleigh wave (RW) mode on Ag(001), temperature-dependent one-dimensional spin-echo scans are performed along this tilt direction. This enables extraction of the phonon linewidth as a function of temperature and thus determination of the intrinsic lifetime of the vibrational mode. As described in the main text, surface phonon energies were then determined from spin-echo measurements over a range of spin-echo times, capturing both real and imaginary components of the polarisation. The solenoid current was scanned symmetrically from -1 A to $+1$ A with 1025 points (Figure 1(b), top). Oscillations in the polarisation indicate periodic vibrational motion of surface atoms and correspond to specific phonon modes. Fourier transformation into the wavelength domain and conversion to energy yields spectra analogous to time-of-flight measurements, with loss and gain peaks reflecting phonon creation and annihilation, respectively (Figure 1(b), bottom). Temperature-dependent measurements of the RW phonon were carried out between 135 and 750 K. As shown in the inset of Figure 1(b), the RW peak and the neighbouring longitudinal resonance (LR) were fitted using two Gaussian functions. Since the focus of the present work is the linewidth of the RW mode at $\Delta K = 0.15$ Å^{-1}, this fitting procedure was applied consistently across the full temperature series. The complete temperature-dependent dataset is displayed in Figure S1(b). Finally, as discussed in the main text, the experimentally observed linewidth contains contributions from both the intrinsic phonon lifetime and instrumental broadening, the latter given by angular broadening in the present case. The angular broadening of the apparatus is estimated based on the angular broadening of the specular peak as shown in Figure S2(a). Using this angular broadening together with the phonon dispersion in the scan curve equation Equation 3 gives an energy broadening (FWHM) $\Delta E_{\mathrm{app}} = 30$ μeV due to the apparatus. The "natural" linewidth of the phonon mode is then given via $(\Gamma_{\mathrm{RW}})^2 = (\Gamma_{\mathrm{total}})^2 - (\Gamma_{\mathrm{app}})^2$ where Γ_{total} is the total energy broadening as determined from the measurements (Figure S1(b)).

As shown in Figure S2(b), systematic deviations from linear behaviour are observed for both the phonon energy and the

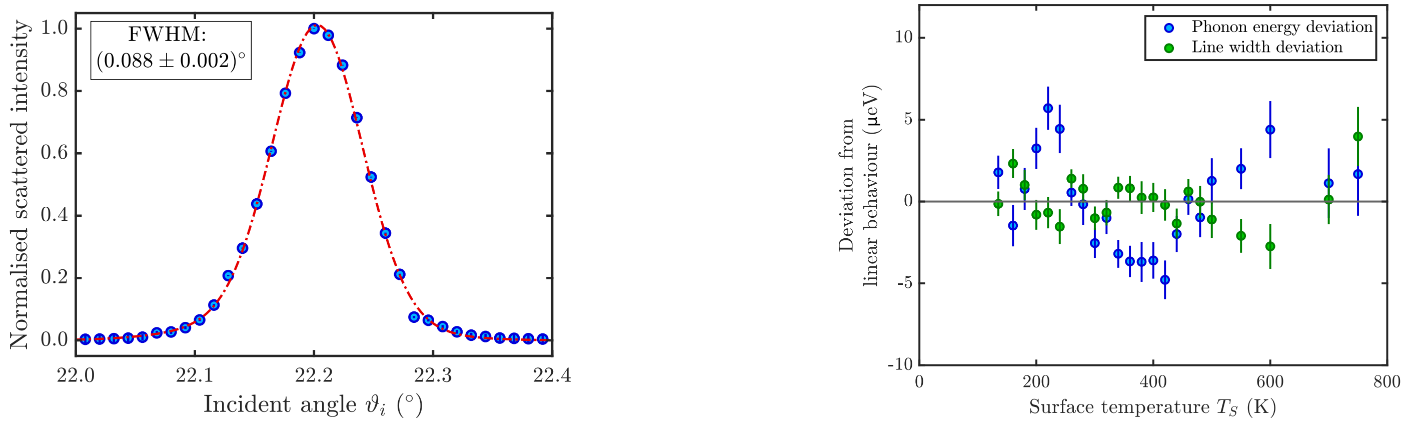

(a) Measured angular broadening of the specular peak for scattering of Ag(001).

(b) A plot of the deviations from the linear fit shows that there seem to be systematic differences with respect to the linear behaviour both for the phonon energy as well as for the linewidth of the RW mode.

Figure S2 Additional scattering characteristics and fit deviation analysis for Ag(001).

linewidth of the RW mode. For the energy, this suggests that a simple anharmonicity model may not fully capture all temperature-dependent effects. Regarding the linewidth, although e-ph interactions can vary significantly with temperature, as demonstrated by[R5], the effect appears less pronounced in the present system. Phonon-phonon interactions are also expected to contribute to linewidth broadening, typically increasing approximately linearly with temperature; however, this depends on the specific phonon decay channels and the involvement of multiphonon processes. In the current case, both deviations remain relatively minor, underscoring the suitability of Ag(001) as a model system for surface phonon studies, while also motivating more detailed investigations in future work.

Supplementary References

[R1] A. Jones, A. Tamtögl, I. Calvo-Almazán and A. Hansen, *Sci. Rep.*, 2016, **6**, 27776.
[R2] P. R. Kole, A. P. Jardine, H. Hedgeland and G. Alexandrowicz, *J. Phys.: Cond. Matt.*, 2010, **22**, 304018.
[R3] A. Jardine, H. Hedgeland, G. Alexandrowicz, W. Allison and J. Ellis, *Prog. Surf. Sci.*, 2009, **84**, 323.
[R4] G. Alexandrowicz and A. P. Jardine, *J. Phys.: Cond. Matt.*, 2007, **19**, 305001.
[R5] B. Liu, W. Allison, B. Peng, N. Avidor, B. Monserrat and A. P. Jardine, *Phys. Rev. Lett.*, 2024, **132**, 176202.
